# Singular Development of an Artery of the Gums

**Published:** 1853-07

**Authors:** 


***Singular Development of an Artery of the Gums***—In the early part of June last, a lady about forty-five years of age, applied to us to have the left superior lateral incisor extracted, the crown of which was nearly destroyed by caries, and the root so much funneled out as to preclude the use of it as a means of support for an artificial tooth. The separation of the gum from the posterior part of the neck of the tooth, was followed by a most extraordinary gush of arterial blood, which escaped by jets in a stream the size of a common knitting needle. The application of pressure with the end of the finger on the gum and against the neck of the tooth and edge of the alveolus arrested the hemorrhage, but on removing it at the expiration of five minutes, the blood spirted as freely as at first. The pressure was reapplied and continued for about thirty minutes, when it was again removed. This time, there was no recurrence of the hemorrhage, a coagulum having formed in the mouth of the wounded artery.

